# Immune activation induced by FOLR2^+^ decidual macrophage deficiency impairs decidualization and angiogenesis in spontaneous abortion

**DOI:** 10.3389/fimmu.2026.1852410

**Published:** 2026-06-23

**Authors:** Si-Man Chen, Meng-Ying Li, Yi-Xing Yang, Nan Liu, Xiao-Yan Cao, Hong-Bo Zhao, Xiao-Yong Zhu, Ming-Qing Li, Feng Xie

**Affiliations:** 1 Obstetrics & Gynecology Hospital of Fudan University, Shanghai Key Lab of Reproduction and Development, Shanghai Key Lab of Female Reproductive Endocrine Related Diseases, Shanghai, China; 2 Department of Reproductive Immunology, The International Peace Maternity and Child Health Hospital, School of Medicine, Shanghai Jiao Tong University, Shanghai, China; 3 Shanghai Key Laboratory of Embryo Original Diseases, Shanghai, China; 4 Medical Center of Diagnosis and Treatment for Cervical Diseases, Obstetrics & Gynecology Hospital of Fudan University, Shanghai, China

**Keywords:** decidualization, FOLR2, immune regulation, macrophage, recurrent spontaneous abortion (RSA), reproductive failure

## Abstract

**Background:**

Decidual macrophages maintain immune tolerance and support decidualization at the maternal–fetal interface. However, the heterogeneity and functions of macrophage subsets in recurrent spontaneous abortion (RSA) remain unclear.

**Methods:**

We integrated single-cell RNA sequencing of human endometrium and decidua with clinical validation. Functional assays using THP-1–derived macrophages and the co-culture with decidual stromal cells were performed to characterize macrophage subsets and investigate their roles in decidualization and RSA pathogenesis.

**Results:**

A subset of FOLR2^+^ macrophages enriched in the decidua showed an M2-like immunoregulatory phenotype. In RSA decidua, FOLR2 expression and the proportion of FOLR2^+^ macrophages were markedly reduced, accompanied by increased inflammatory signaling and impaired angiogenic and adhesion interactions. Functional assays showed that FOLR2 overexpression enhanced immunoregulatory, tissue-resident, and pro-angiogenic properties, whereas FOLR2 silencing promoted inflammatory responses and impaired angiogenesis. Reciprocal crosstalk between DSCs and FOLR2^+^ macrophages established a positive feedback loop that promoted stromal decidualization and supported pregnancy maintenance.

**Conclusions:**

FOLR2^+^ macrophages represent an immunoregulatory subset at the maternal–fetal interface with tissue-resident and pro-angiogenic features. The loss of FOLR2^+^ macrophages in RSA may contribute to immune dysregulation and defective decidualization, providing new insights into the immune mechanisms underlying RSA pathogenesis.

## Introduction

Spontaneous abortion (SA) affects approximately 8–15% of clinically recognized pregnancies, with more than 80% occurring during the first trimester (1). Among these women, approximately 5–10% experience recurrent spontaneous abortion (RSA), defined as the loss of two or more clinically recognized pregnancies before 20 weeks of gestation (2). In addition to compromised reproductive potential, women with RSA are at increased risk of gynecological disorders, long-term health complications, and adverse outcomes in subsequent pregnancies (3).

The etiology of RSA is multifactorial, encompassing both maternal and embryonic factors. Maternal contributors include age-related, anatomical, infectious, and endocrine abnormalities, whereas embryonic causes are largely attributed to chromosomal defects (4–6). Nevertheless, nearly half of cases remain unexplained despite thorough clinical evaluation, underscoring critical gaps in our understanding of the underlying mechanisms. Elucidating the molecular and cellular basis of RSA is critical for improving risk assessment and identifying potential therapeutic targets.

The maternal–fetal interface consists primarily of embryo-derived trophoblasts, maternal decidual stromal cells (DSCs), and decidual immune cells (DICs). Decidualization is the progesterone-driven differentiation of endometrial stromal cells into decidual stromal cells, which undergo morphological and transcriptional changes and acquire secretory and immunoregulatory functions to support embryo implantation and early pregnancy (7). The establishment and maintenance of pregnancy depend on coordinated trophoblast function, proper decidualization, regulated immune cell recruitment, and precise maternal–fetal crosstalk (8). Dysregulation of any of these components may compromise early pregnancy and predispose to later obstetric complications, including intrauterine growth restriction and preeclampsia (9). Under physiological conditions, the decidual immune compartment is predominantly composed of natural killer (NK) cells, macrophages, and T cells, with smaller populations of dendritic cells (10, 11). Increasing evidence indicates that patients with miscarriage exhibit abnormal immune cell phenotypes in both peripheral blood and the decidua. Aberrant immune cell infiltration, disturbed cellular composition, and impaired immunoregulatory capacity are considered critical contributors to pregnancy failure (12).

DICs account for approximately 40–50% of the cellular composition at the maternal–fetal interface. Among them, decidual macrophages (dMac) constitute nearly 20% of the immune compartment during early pregnancy, second only to NK cells (13). The maintenance of dMac homeostasis is crucial for successful pregnancy, as these cells orchestrate multiple processes essential for gestational progression, including promoting trophoblast invasion, facilitating spiral artery remodeling and placental angiogenesis, and modulating maternal–fetal immune responses (14). In later pregnancy, dMac also contribute to cervical ripening and the initiation of labor (15). Experimental depletion of macrophages in pregnant mouse models leads to fetal resorption, highlighting their indispensable role in pregnancy maintenance (16). Dysregulation of dMac function has been associated with a range of pregnancy-related complications, including miscarriage, intrauterine growth restriction, and preeclampsia (17).

Macrophages are classified as classically activated (M1) or alternatively activated (M2) macrophages (18). M1 polarization is typically induced by lipopolysaccharide (LPS) or interferon-γ (IFN-γ) and is characterized by the production of pro-inflammatory cytokines, including interleukin-1 beta (IL-1β) and tumor necrosis factor-α (TNF-α), thereby mediating pathogen clearance and inflammatory responses (19). In contrast, M2 macrophages are primarily induced by IL-4 and IL-13 and express anti-inflammatory mediators such as IL-10, transforming growth factor-beta (TGF-β), and macrophage colony-stimulating factor (M-CSF), promoting inflammation resolution, apoptotic cell clearance, tissue remodeling, and immune homeostasis (20). However, recent studies suggest that macrophage functional states are not strictly confined to the M1/M2 dichotomy but are dynamically regulated by integrated microenvironmental signals, resulting in a continuum of activation states with mixed or transitional phenotypes rather than discrete polarization subsets (21, 22). In this context, M1- and M2-like annotations are commonly applied in a functional sense to describe macrophage states associated with pro-inflammatory and immunoregulatory programs, respectively, without implying rigid polarization boundaries. During early pregnancy, a transient pro-inflammatory milieu at the implantation window is associated with a relative increase in M1-like activity. However, throughout most of gestation, dMac predominantly exhibit an M2-like phenotype, supporting maternal–fetal tolerance and tissue remodeling (23).

Folate Receptor 2 (FOLR2) is predominantly expressed on M2-like macrophages and has emerged as a reliable marker for identifying specific M2 subpopulations. Previous studies have identified FOLR2^+^ macrophages (FOLR2^+^ Mac) as a distinct subset of tissue-resident macrophages (TRMs) among various cancers, including breast cancer and lung adenocarcinoma (24, 25). These cells often exhibit perivascular localization, suggesting a key role in regulating the local angiogenic niche and vascular remodeling (26). Previous single-cell studies have identified FOLR2 as a marker of macrophages with an anti-inflammatory phenotype in the human decidua (27). However, their precise contributions to angiogenesis and the maintenance of a healthy pregnancy remain unclear. Furthermore, the specialized intercellular crosstalk between FOLR2^+^ Mac and other decidual cell types at the maternal–fetal interface has yet to be elucidated.

In this study, we identified a subset of FOLR2^+^ Mac within the human decidua and systematically characterized their tissue-resident features, adhesion-associated properties, and immunotolerant phenotype. We further explored their functional contributions to angiogenesis and the decidualization of DSCs. Notably, we explored the clinical association between the reduction of FOLR2^+^ Mac and RSA, suggesting that this macrophage subset may represent a potential biomarker candidate and a possible target for future therapeutic investigation in early pregnancy loss.

## Materials and methods

### Clinical samples

All procedures involving human participants were approved by the Human Research Ethics Committee of the Obstetrics and Gynecology Hospital of Fudan University (approval no. 2023-136). Written informed consent was obtained from all participants prior to tissue collection. Decidual tissues were collected from women with normal pregnancy (NP) or RSA. The control group consisted of women with clinically normal early-stage pregnancies who underwent elective termination for non-medical reasons (n = 10; age range, 20–35 years). The RSA group included women diagnosed with RSA, defined as the loss of two or more clinically recognized pregnancies before 20 weeks of gestation (n = 7; age range, 20–35 years). All pregnancies were confirmed by ultrasound examination and serum human chorionic gonadotropin (hCG) measurement. Women with RSA attributable to endocrine disorders, anatomical abnormalities, genetic defects, or infection were excluded from the study. Baseline characteristics of RSA patients and control subjects are listed in **Supplementary Table 1**.

### Single-cell RNA sequencing acquisition and processing

Raw scRNA-seq data of human endometrium and decidua were obtained from our previous studies deposited in the NCBI Gene Expression Omnibus (GEO) under accession numbers GSE183837 (endometrium) (28) and GSE194219 (decidua) (29). In addition, scRNA-seq data from first-trimester decidual tissues, including five normal samples and three RSA samples, were retrieved from GEO (GSE214607) (30).

Data processing and analysis were conducted using the Seurat R package (version 4.3.0.1) in R (version 4.2.3). Quality control filtering was applied to exclude low-quality cells. Cells expressing 200–5,000 genes and containing less than 15% mitochondrial transcripts were retained for downstream analysis. The 2,000 most variable genes were identified for principal component analysis (PCA). To correct for batch effects across individual samples and experimental conditions, the Harmony algorithm (31) was applied to the PCA embeddings. Harmony-corrected principal components were then used for downstream clustering and visualization.

Graph-based clustering was performed using Seurat’s FindClusters function (resolution = 0.5; k-nearest neighbors = 10). Uniform manifold approximation and projection (UMAP) was applied for nonlinear dimensionality reduction and visualization of cell clusters. Differentially expressed genes (DEGs) among clusters were identified using the Wilcoxon rank-sum test implemented in Seurat’s FindAllMarkers function. Cell type annotation was performed according to the expression of canonical marker genes, with reference to published literature and the CellMarker database.

Overall, all sequenced endometrial and decidual cells in GSE183837 and GSE194219 were classified into seven major cell types based on canonical marker gene expression: fibroblast-like stromal cells (SC; HOXA10, MME, COL1A1), NK cells (PTPRC, NCAM1, NKG7), macrophages (Mac; PTPRC, CD14, CD68), T cells (PTPRC, CD3D), B cells (CD79A, CD79B), endothelial cells (Endo; PECAM1, CDH5), and epithelial cells (Epi; KRT7, KRT8, KRT18, EPCAM).

Decidual cells in GSE214607 were further annotated into ten major cell types based on canonical markers: decidual stromal cells (DSC; DKK1, HAND2, COL1A1), perivascular cells (PV; COL1A1, ACTA2, RGS5), endothelial cells (Endo; CDH5, PECAM1), epithelial cells (Epi; EPCAM, KRT7, KRT8), extravillous trophoblasts (EVT; KRT7, KRT8, HLA-G, NOTUM), NK cells (NCAM1, PTPRC, NKG7), macrophages (Mac; PTPRC, CD14, CD68), T cells (PTPRC, CD3D), dendritic cells (DC; PTPRC, CLEC9A, XCR1), and neutrophils (CMTM2, CXCR2).

### Identification and analysis of FOLR2^+^ macrophages

Macrophages were divided into FOLR2^+^ and FOLR2⁻ subsets according to FOLR2 expression levels. Cells with FOLR2 expression above the median value within the macrophage population were classified as FOLR2^+^ Mac, whereas those at or below the median were defined as FOLR2⁻ Mac. Subsequent analyses were performed to compare transcriptional profiles and functional characteristics between the two subsets.

### Kyoto encyclopedia of genes and genomes and gene ontology enrichment analysis

KEGG and GO enrichment analyses were conducted on the top 50 marker genes of FOLR2^+^ Mac using the ClusterProfiler R package (version 4.14.6). Pathways with a *p* value < 0.05 were considered statistically significant. The expression patterns of genes involved in representative enriched pathways were visualized using dot plots to illustrate pathway-associated gene expression across cell populations.

### Cell-cell communication analysis using CellChat

Cell–cell communication analysis was performed using the CellChat R package (version 1.6.1) to infer intercellular signaling interactions between FOLR2^+^ Mac and other decidual cell populations in control and RSA samples. The analysis was conducted based on a curated database of known ligand–receptor interactions. Communication probabilities were calculated using the computeCommunProb function, which estimates both the number and strength of potential interactions between defined cell types. To characterize differential signaling patterns, FOLR2^+^ Mac was designated as the signaling source, and the netVisual_bubble function was applied to visualize significantly upregulated and downregulated ligand–receptor pairs (*p* < 0.01) between groups.

### Primary cell isolation

Primary endometrial immune cells (EICs), DSCs, and DICs were isolated from human endometrial and decidual tissues as previously described (29). Briefly, endometrial and decidual tissues were rinsed thoroughly with PBS, sectioned into 1 mm3 pieces and digested with 1 mg/ml collagenase type IV (Sigma-Aldrich; 9003–98-9) for 30 min at 37 °C. Following enzymatic digestion, cell suspensions were filtered sequentially through sterile gauze filters (100-, 200-, and 400-mesh pore sizes) to remove undigested tissue fragments. The filtrates were centrifuged at 1,500 rpm for 8 min, and the resulting cell pellets were resuspended in DMEM/F12 medium. Cells were then layered onto a discontinuous Percoll density gradient (20%, 40%, and 60%; Percoll bulk standard) and centrifuged at 2,500 rpm for 30 min. DSCs were collected from the 20%/40% interface, whereas EICs and DICs were harvested from the 40%/60% interface.

DSCs were cultured in complete DMEM/F-12 medium (Gibco, Thermo Fisher Scientific; 11330033) supplemented with 10% fetal bovine serum (FBS; Gibco; A5669701) and 1% penicillin–streptomycin–amphotericin B (NCM Biotech; C125C8). DICs were cultured in complete RPMI-1640 medium (GENOM; GNM31800) supplemented with 10% FBS and 1% penicillin–streptomycin–amphotericin B. Cells were maintained in a humidified incubator at 37 °C with 5% CO₂ and 20% O₂.

### Flow cytometry

Fluorochrome-conjugated anti-human antibodies used for flow cytometry included: APC anti-human Folate Receptor β (FOLR2) antibody (BioLegend; clone 94b/FOLR2), FITC anti-human CD14 antibody (BioLegend; clone M5E2), PE anti-human CD45 antibody (BioLegend; clone QA21A24), APC/Cyanine7 anti-human CD14 antibody (BioLegend; clone HCD14), APC anti-human CD86 antibody (BioLegend; clone BU63), and PE anti-human CD206 (MMR) antibody (BioLegend; clone 15-2). For experiments involving lentiviral transduction with GFP-expressing constructs, APC/Cy7-conjugated CD14 antibody was used to avoid spectral overlap with GFP fluorescence in the FITC channel.

Surface staining was performed in staining buffer (PBS containing 2% FBS) for 20 min at room temperature in the dark. For intracellular staining, cells were fixed and permeabilized using the Cyto-Fast™ Fix/Perm Buffer Set (BioLegend) according to the manufacturer’s instructions, followed by antibody incubation for 20 min at room temperature in the dark. Cells were washed three times and resuspended in PBS prior to acquisition. Data were acquired on a CytoFLEX flow cytometer (Beckman Coulter, Brea, CA, USA). Compensation was performed using single-stained controls, and fluorescence-minus-one (FMO) controls were used to define gating boundaries. The gating strategy was applied consistently across all samples based on FMO controls and clear separation of negative and positive populations on the log-scale fluorescence intensity axis. The same gating strategy and relative thresholds were maintained across all experimental batches. Data were analyzed using FlowJo software (BD Biosciences; version 10.8.1).

### Immunofluorescence

Human endometrial tissues were obtained from women in the control group, while decidual tissues were collected from both control and RSA patients. All samples were fixed in 4% paraformaldehyde, paraffin-embedded, and sectioned into 4-μm-thick slices. Sections were baked at 60 °C for 2 h, deparaffinized in xylene, and rehydrated through a graded ethanol series. Antigen retrieval was performed by boiling sections in Tris–EDTA buffer (pH 9.0; C1038, Solarbio, Beijing, China) for 10 min, followed by cooling to room temperature. After washing with PBS, sections were blocked with 5% bovine serum albumin (BSA) for 1 h at room temperature. Sections were incubated overnight at 4 °C with the following primary antibodies: rabbit anti-FOLR2 (1:100; 31264, Proteintech, Wuhan, China) and mouse anti-CD68 (1:200; 14-0688-82, Invitrogen). After washing, sections were incubated with species-appropriate fluorescent secondary antibodies for 1 h at room temperature in the dark. Nuclei were counterstained with DAPI (C0065, Solarbio). Images were acquired using an epifluorescence microscope (Olympus Corporation, Tokyo, Japan; BX53). Fluorescence-positive areas were quantified using ImageJ software (National Institutes of Health; version 1.8.0).

### Cell lines

THP-1 cells were cultured in complete RPMI-1640 medium (GENOM; GNM31800) supplemented with 10% FBS and 1% penicillin–streptomycin–amphotericin B. Cells were maintained in a humidified incubator at 37 °C with 5% CO₂ and 20% O₂. THP-1 cells were differentiated into M0 macrophages by treatment with 100 ng/mL phorbol 12-myristate 13-acetate (PMA) for 48 h, as previously described (32).

Human umbilical vein endothelial cells (HUVECs) were cultured in complete DMEM/F12 medium (Gibco; 11330033) supplemented with 10% FBS and 1% penicillin–streptomycin–amphotericin B and maintained in a humidified incubator at 37 °C with 5% CO₂ and 20% O₂.

### Quantitative reverse transcription–polymerase chain reaction

Total RNA was extracted from cells using the EZ-press RNA purification kit (EZ Bioscience; ZScience Biotechnology Corporation, Roseville, USA; B0004DP) according to the manufacturer’s instructions. Complementary DNA (cDNA) was synthesized using a reverse transcription kit (Yeasen Biotech, Shanghai, China; 11141ES60). qRT-PCR was performed using qPCR SYBR Green Master Mix (Yeasen Biotech; 11184ES08). Relative mRNA expression levels were calculated using the 2^−ΔΔCt^ method. ACTB was used as the internal reference gene. All reactions were performed in triplicate. The sequences of the primers used in this study are listed in **Supplementary Table 2**.

### Lentiviral transduction

THP-1 cells (5 × 10⁵ cells/well) were seeded in 6-well plates in complete RPMI-1640 medium and transduced with FOLR2-overexpressing lentivirus (OE-FOLR2; PGMLV-CMV-H_FOLR2-3×Flag-EF1-ZsGreen1-T2A-Puro) or negative control lentivirus (PGMLV-CMV-EF1-ZsGreen1-T2A-Puro) (Genomeditech Co., Ltd., Shanghai, China) at a multiplicity of infection (MOI) of 30. The culture medium was replaced with fresh complete medium after 12 h. Transduction efficiency was assessed by fluorescence microscopy (Olympus Corporation) 48 h after infection. The expression level of FOLR2 was further verified by western blotting at 72 h post-transduction. Stable FOLR2-overexpressing THP-1 cells were selected using puromycin (2 mg/L).

### siRNA transfection

Negative control and FOLR2-specific siRNA oligonucleotides were designed and synthesized by Genomeditech (Shanghai, China). THP-1 cells were transfected with siRNA oligonucleotides using CALNP™ RNAi *in vitro* (D-Nano Therapeutics, Beijing, China; DN001) according to the manufacturer’s instructions. After 48 h of transfection, FOLR2 knockdown efficiency was evaluated by western blot analysis. The sequences of the siRNA oligonucleotides used in this study are listed in **Supplementary Table 3**.

### Western blotting

NC or OE-FOLR2 THP-1 cells, as well as DSCs, were washed with PBS and lysed in radioimmunoprecipitation assay (RIPA) lysis buffer (Beyotime, Shanghai, China; P0013B) supplemented with 1% protease inhibitor cocktail (Yeasen Biotech; 20124ES03). The lysates were centrifuged at 10,000 × g for 20 min at 4 °C, and the supernatants were collected for protein analysis. Protein concentrations were determined using a BCA Protein Assay Kit (Beyotime; P0012). Equal amounts of protein (20 μg per sample) were separated by 10% sodium dodecyl sulfate–polyacrylamide gel electrophoresis (SDS–PAGE) and subsequently transferred onto polyvinylidene fluoride (PVDF) membranes (0.45 μm, EMD Millipore). The membranes were blocked with 5% skimmed milk for 1 h at room temperature and then incubated overnight at 4 °C with the following primary antibodies: anti-FOLR2 (Proteintech; 31264), anti-IGFBP1 (Abcam; ab181141), anti-PRL (Abcam; ab110642), anti-β-actin (Proteintech; 112544), and anti-β-tubulin (Cell Signaling Technology; 2146). β-actin and β-tubulin were used as internal loading controls. The membranes were washed three times with TBST (0.1% Tween-20 in Tris-buffered saline) and incubated with HRP-conjugated secondary antibodies (goat anti-rabbit IgG-HRP, Cell Signaling Technology; 7074, or horse anti-mouse IgG-HRP, Cell Signaling Technology; 7076) for 1 h at room temperature. Protein bands were visualized using an enhanced chemiluminescence (ECL) detection kit (Yeasen Biotech; 36208ES60) and quantified using ImageJ software (National Institutes of Health; version 1.8.0). Protein expression levels were determined by densitometric analysis of band intensities and normalized to β-actin (ACTB). Each Western blot experiment was performed and analyzed independently.

### Enzyme-linked immunosorbent assay

The culture supernatants of NC and OE-FOLR2 THP-1 cells were collected after 48 h of stimulation with 100 ng/mL PMA and centrifuged at 12,000 × g for 15 min at 4 °C to remove cellular debris. The concentrations of TNF-α and IL-10 in the supernatants were measured using a Human TNF-α ELISA Kit (Elabscience, Wuhan, China; E-OSEL-H0005) and a Human IL-10 ELISA Kit (Elabscience; E-EL-H6154), respectively, according to the manufacturer’s instructions.

### Co-culture

A Transwell co-culture system (Corning, NY, USA) was used to investigate the interactions between THP-1-derived macrophages and HUVECs or DSCs.

THP-1 cells from the NC, OE-FOLR2, or siFOLR2 groups were first seeded into Transwell inserts (upper chambers) at a density of 5 × 10⁵ cells per well and treated with 100 ng/mL PMA for 24 h to induce macrophage differentiation and promote cell adherence. Meanwhile, HUVECs or DSCs were seeded in the lower chambers of 6-well plates at a density of 5 × 10⁵ cells per well and cultured overnight at 37 °C with 5% CO₂. After 24 h of pretreatment, the Transwell inserts containing THP-1-derived macrophages were placed into the wells containing HUVECs or DSCs to initiate co-culture. After 48 h of co-culture, cells in the lower chambers were collected for subsequent experiments.

For the DSC induction experiments, DSCs were first seeded into Transwell inserts (upper chambers) at a density of 3 × 10^5^ cells per well and cultured overnight. Meanwhile, THP-1 cells were seeded in the lower chambers at a density of 1 × 10^6^ cells per well and treated with 100 ng/mL PMA for 24 h to induce macrophage differentiation. After pretreatment, the Transwell inserts containing DSCs were placed into the wells containing THP-1-derived macrophages to initiate co-culture. After 48 h of co-culture, THP-1 cells in the lower chambers were collected for subsequent analysis.

### Tube formation assay

HUVECs were cultured in 6-well plates and co-cultured following the procedure described previously. Matrigel (Corning, NY, USA; 356234), previously dissolved overnight at 4 °C, was added to 24-well plates and allowed to solidify at 37 °C for 30 min. HUVECs were adjusted to a concentration of 1.5 × 10^5^ cells/mL and seeded onto the solidified Matrigel in 24-well plates, then cultured at 37 °C under 5% CO2. Tube formation was observed at the 12-hour time point. The numbers of nodes, tubes, and meshes were calculated by ImageJ software.

### Statistical analysis

Each experiment included three biological replicates per condition and was independently repeated at least three times. Data are presented as the mean ± standard error of the mean (SEM), where n represents the number of independent experiments. Statistical analyses were performed using GraphPad Prism software (version 10.1.2; GraphPad Software Inc., San Diego, CA, USA). Data were first assessed for normality. The specific statistical tests used are indicated in the figure legends. For comparisons between two groups, an unpaired Student’s t-test was used when data followed a normal distribution; otherwise, the nonparametric Mann–Whitney U test was performed. For comparisons among more than two groups under a single condition, one-way analysis of variance (ANOVA) was applied. A *p* value < 0.05 was considered statistically significant. Exact *p* values are reported in the figures.

## Results

### Identification of FOLR2^+^ macrophages enriched in the decidua

**Figure 1:**
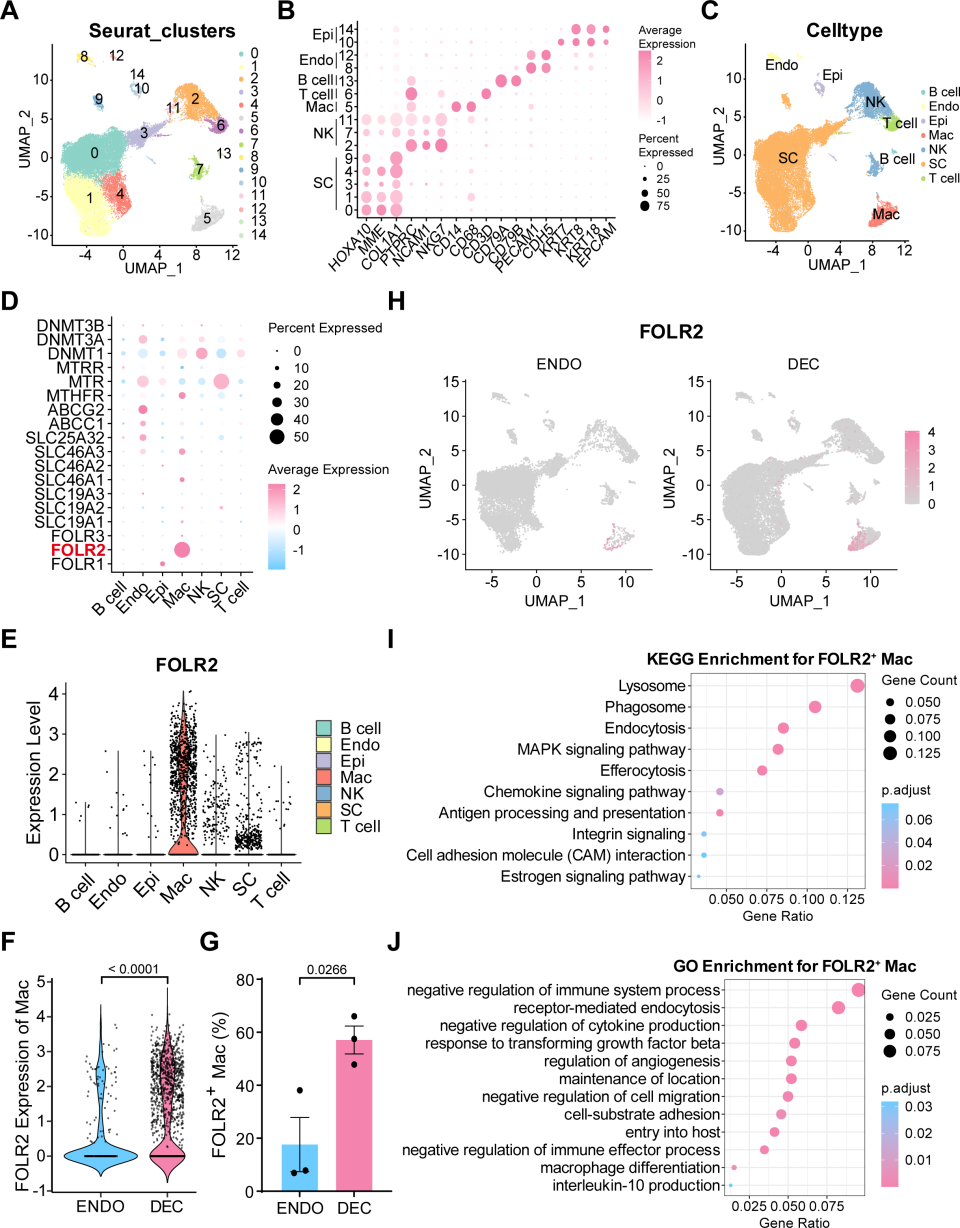
Increased FOLR2^+^ Macrophages in the Decidua. **(A)** UMAP plot showing 15 identified cell types from single-cell transcriptomes of human endometrium and decidua. Each dot represents a single cell, colored by cell type. **(B)** Dot plot of representative marker genes used to define each cell type. **(C)** UMAP visualization of seven major cell populations: SC, Mac, NK cells, Epi, Endo, T cells, and B cells. **(D)** Dot plot showing the expression of genes involved in folate metabolism across the seven cell populations. **(E)** Violin plot of FOLR2 expression across the seven cell populations. **(F)** Violin plot comparing FOLR2 expression in macrophages from endometrium (ENDO) and decidua (DEC). **(G)** Percentage of FOLR2^+^ Mac in ENDO and DEC. **(H)** Feature plot showing FOLR2 expression in ENDO and DEC. **(I)** KEGG pathway enrichment analysis of the top 50 marker genes in FOLR2^+^ Mac from ENDO and DEC. **(J)** GO enrichment analysis of the top 50 marker genes in FOLR2^+^ Mac from ENDO and DEC. Data are presented as mean ± SEM and analyzed using Student’s *t*-test.

To evaluate the heterogeneity and dynamic changes of dMac, we reanalyzed our previously generated scRNA-seq datasets of the human endometrium during the proliferative and secretory phases (GSE183837) (28) and the decidualized endometrium during early pregnancy (GSE194219) (29). Based on canonical marker gene expression, all endometrial and decidual cells were classified into seven major cell populations: stromal cells (SC), NK cells, macrophages (Mac), T cells, B cells, endothelial cells (Endo), and epithelial cells (Epi) (**Figures 1A–C**; see Methods). For instance, SC expressed HOXA10, MME, and COL1A1. Mac expressed PTPRC, CD14, and CD68, whereas NK cells expressed PTPRC, NCAM1, and NKG7 (**Figure 1B**).

Among genes involved in folate metabolism, FOLR2 was selectively expressed in macrophages and showed the highest expression levels (**Figures 1D, E**). Notably, dMac exhibited significantly higher FOLR2 expression compared with macrophages from the normal endometrium (**Figure 1F**). Consistently, the proportion of FOLR2^+^ Mac (defined as macrophages with FOLR2 expression above the median level) was markedly increased in the decidua (**Figures 1G, H**), suggesting a potential role for this macrophage subset during decidualization.

To further investigate the functional characteristics of FOLR2^+^ Mac, enrichment analyses were performed. KEGG pathway analysis revealed significant enrichment in pathways associated with endocytosis, integrin signaling, cell adhesion molecule (CAM) interactions, and estrogen signaling. In parallel, GO analysis indicated enrichment in biological processes including negative regulation of immune system processes, response to transforming growth factor-β, regulation of angiogenesis, maintenance of localization, macrophage differentiation, and interleukin-10 production.

As FOLR2^+^ Mac have been reported as tissue-resident macrophages in tumors, we hypothesized that decidual FOLR2^+^ Mac may share similar features, including enhanced adhesion capacity and an immunotolerant phenotype. Functionally, these cells may contribute to anti-inflammatory and pro-angiogenic functions, thereby facilitating decidualization and successful embryo implantation.

### Impaired FOLR2^+^ macrophage function in RSA decidua

**Figure 2:**
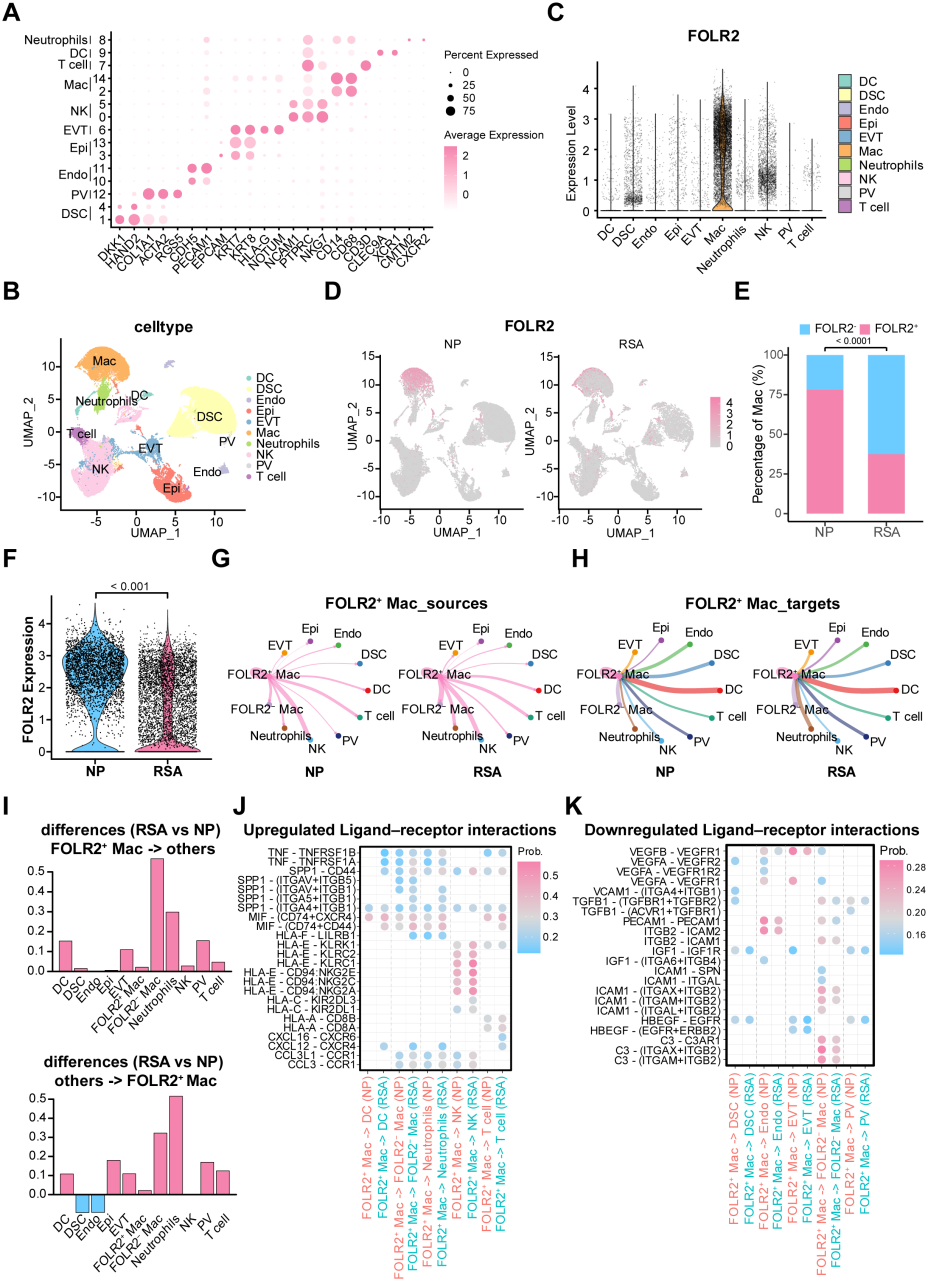
Decreased FOLR2^+^ macrophages and impaired function in RSA decidua. **(A)** Dot plot of representative marker genes used to define each cell type. **(B)** UMAP visualization of ten major cell populations: DSC, Mac, NK cells, Epi, Endo, T cells, EVT, DC, neutrophils, and PV. **(C)** Violin plot showing FOLR2 expression across the ten cell populations. **(D)** Feature plot showing FOLR2 expression in decidual cells from the normal pregnancy (NP) and RSA groups. **(E)** Proportion of FOLR2^+^ Mac in NP and RSA decidua. **(F)** Violin plot showing FOLR2 expression in dMac from NP and RSA groups. **(G)** Circle plots depicting intercellular communication from FOLR2^+^ Mac to other cell types in the NP and RSA groups, analyzed using CellChat. Each node represents a cell type, with node size proportional to the total number of signaling interactions sent or received by that cell type. Edges indicate inferred communications between cell types, and edge width is proportional to the communication weight. Node colors distinguish different cell types and do not imply quantitative values. **(H)** Circle plots depicting intercellular communication from other cell types to FOLR2^+^ Mac in the NP and RSA groups. **(I)** Bar plots showing differences in the number of inferred interactions between NP and RSA groups for signaling from FOLR2^+^ Mac to other cell types (top) and from other cell types to FOLR2^+^ Mac (bottom). **(J)** Dot plot showing upregulated ligand–receptor signaling pathways involving FOLR2^+^ Mac in RSA decidua. **(K)** Dot plot showing downregulated ligand–receptor signaling pathways involving FOLR2^+^ Mac in RSA decidua. Data are presented as mean ± SEM and analyzed using Student’s *t*-test.

Given the potential role of FOLR2^+^ Mac in fostering decidualization and supporting pregnancy maintenance, we next investigated their functional alterations in RSA decidua by analyzing single-cell RNA-sequencing data (GSE214607). Based on canonical marker gene expression, all decidual cells were initially classified into ten major cell types: decidual stromal cells (DSC), perivascular cells (PV), Endo, Epi, extravillous trophoblasts (EVT), NK cells, Mac, T cells, dendritic cells (DC), and neutrophils (**Supplementary Figure 1A**; **Figures 2A, B**; see Methods).

Consistent with observations in normal decidua, FOLR2 expression remained largely restricted to macrophages (**Figure 2C**). Moreover, both the expression levels of FOLR2 and the proportion of FOLR2^+^ Mac were significantly reduced in RSA decidua compared with controls (**Figures 2D–F**), indicating that reduced abundance and functional impairment of FOLR2^+^ Mac may represent a key feature of RSA.

To further characterize intercellular communication, we performed CellChat analysis (**Supplementary Figures 1B, C**). In the RSA microenvironment, FOLR2^+^ Mac showed increased predicted ligand–receptor interactions with FOLR2⁻ Mac, neutrophils, and DCs, along with enrichment of inflammatory signaling pathways, including TNF and SPP1 signaling pathways (**Figures 2G–J**). Conversely, predicted interactions between FOLR2^+^ macrophages and DSCs or endothelial cells were reduced, accompanied by decreased representation of ligand–receptor pairs related to angiogenesis, immunoregulation, and cell adhesion, including VEGF, TGFB, and ITGB2 signaling pathways (**Figures 2G–I, K**). These findings suggest an altered intercellular communication landscape involving FOLR2^+^ macrophages in RSA decidua, characterized by a shift toward pro-inflammatory signaling and reduced predicted interactions associated with angiogenesis and tissue remodeling.

Collectively, these findings indicate that FOLR2^+^ Mac in RSA decidua exhibit impaired pro-angiogenic, immunoregulatory, and adhesive functions, likely compromising decidualization and early pregnancy maintenance.

### Validation of decreased FOLR2^+^ macrophages in RSA decidua

To validate the distribution of FOLR2^+^ Mac in clinical samples, we collected human endometrial tissues, NP decidua, and RSA decidua. Flow cytometry was performed to identify CD45^+^ immune cells, which were further subdivided into CD14^+^ macrophages and CD14⁻ immune cells. Consistent with our single-cell RNA-sequencing analysis, FOLR2 expression was largely restricted to macrophages, with minimal expression detected in other immune cell types.

**Figure 3:**
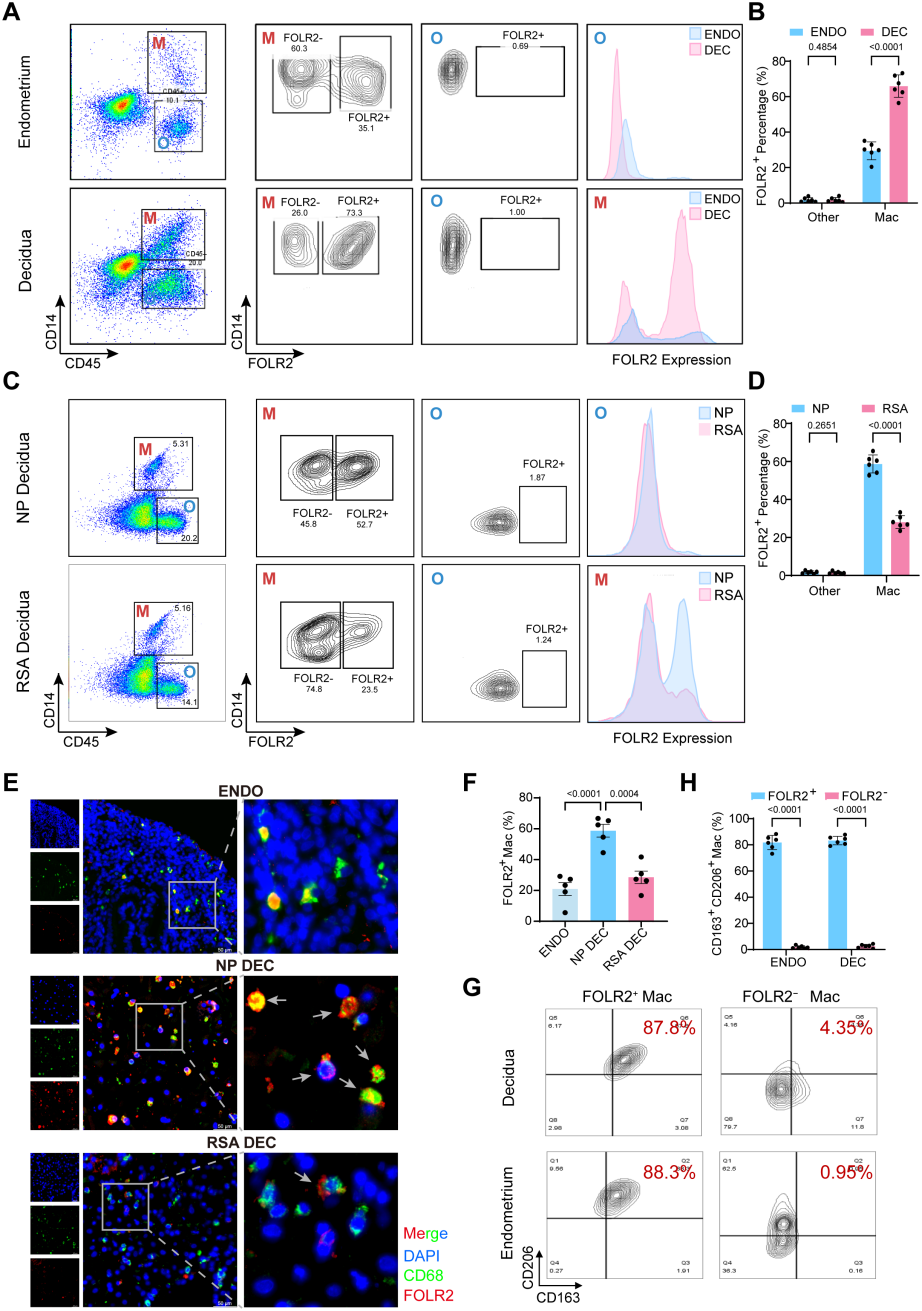
Validation of decreased FOLR2^+^ macrophages with an immunoregulatory phenotype in RSA decidua. **(A)** Flow cytometry analysis of endometrial and decidual cells. CD45^+^ cells were gated to identify immune cells, and CD14^+^ cells were further gated to define macrophages. FOLR2 expression is shown by histogram plots. **(B)** Percentage of FOLR2^+^ cells among other immune cells (O) and macrophages (M) (n = 6). **(C)** Flow cytometry analysis of decidual cells from NP and RSA groups. **(D)** Percentage of FOLR2^+^ cells among other immune cells (O) and macrophages (M) in NP and RSA decidua (n = 6). Flow cytometry gating was performed using FMO and single-stain controls, and the same gating strategy and thresholds were consistently applied across all experimental batches. **(E, F)** Immunofluorescence staining **(E)** and quantitative analysis **(F)** of FOLR2 expression in human endometrium, NP decidua, and RSA decidua. CD68 was used to identify macrophages (n = 5). Scale bars, 50 μm. **(G)** Flow cytometry analysis of CD163 and CD206 expression in FOLR2^+^ and FOLR2⁻ Mac in endometrium and decidua. **(H)** Percentage of CD163^+^CD206^+^ macrophages among FOLR2^+^ and FOLR2⁻ Mac in endometrium and decidua (n = 6). Data are presented as mean ± SEM. Statistical significance was determined using Student’s *t*-test or one-way ANOVA.

Notably, the proportion of FOLR2^+^ Mac was markedly enriched in decidual tissues, accounting for approximately 50–75% of the total dMac (**Figures 3A, B**), further supporting a potential role for this macrophage subset in decidualization. Compared with NP decidua, the proportion of FOLR2^+^ Mac was significantly reduced in RSA decidua, whereas other immune cell populations exhibited minimal FOLR2 expression (**Figures 3C, D**). Immunofluorescence staining further corroborated these findings. In endometrial tissues, CD68^+^ macrophages displayed relatively low FOLR2 expression. In contrast, CD68^+^ macrophages in NP decidua exhibited markedly elevated FOLR2 levels. However, RSA decidual tissues showed a decreased abundance of FOLR2^+^ Mac, consistent with the flow cytometry results (**Figures 3E, F**).

Furthermore, flow cytometric analysis revealed that FOLR2^+^ Mac predominantly exhibited an anti-inflammatory phenotype, as indicated by a higher proportion of CD163^+^CD206^+^ macrophages. In contrast, FOLR2⁻ Mac showed a significantly lower frequency of CD163^+^CD206^+^ cells, suggesting a shift toward a more pro-inflammatory phenotype (**Figures 3G, H**). Together, these clinical data validate our single-cell findings and highlight a marked reduction of FOLR2^+^ Mac in RSA decidua.

### FOLR2-overexpressing macrophages promote an immunoregulatory and pro-angiogenic phenotype

**Figure 4:**
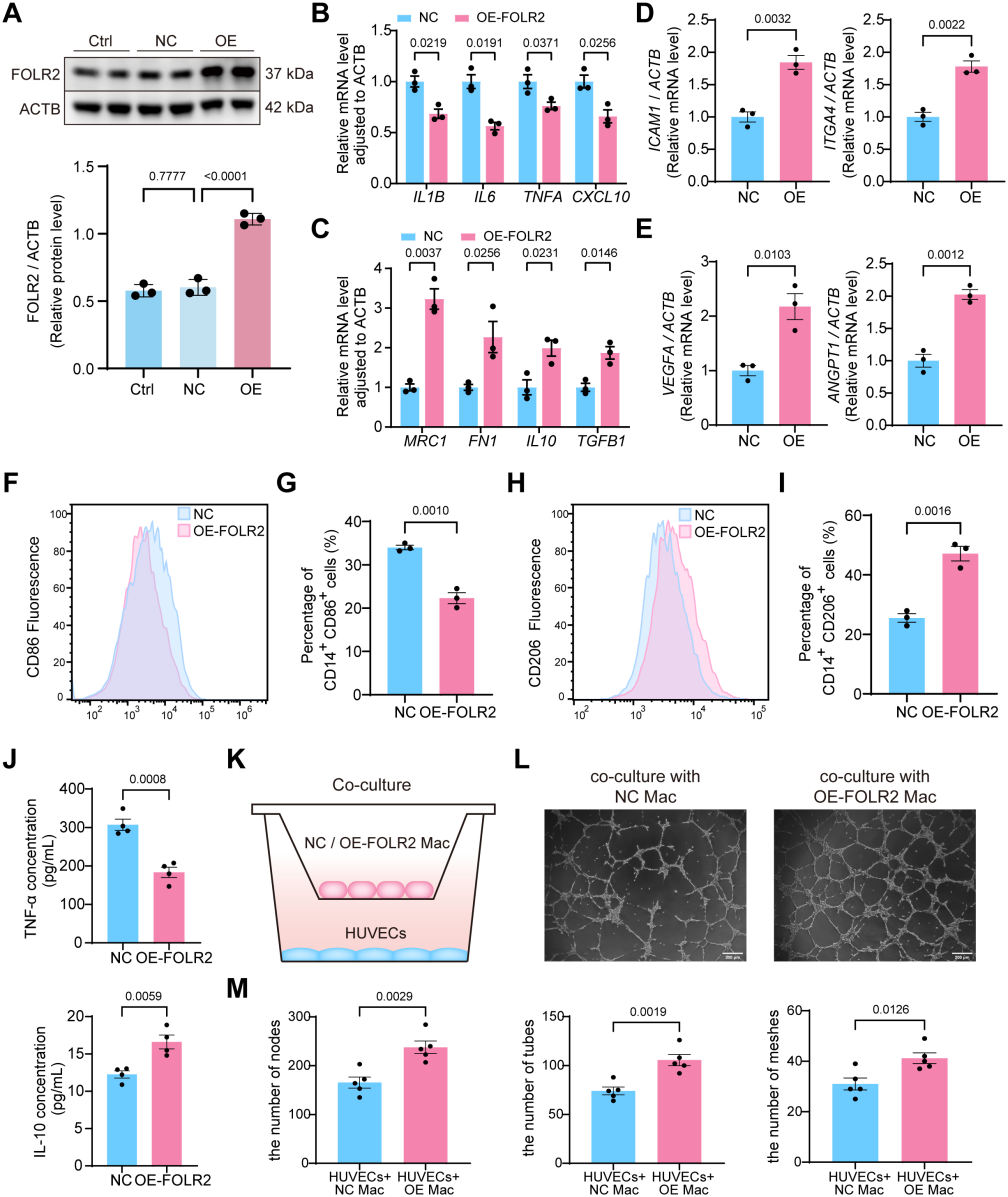
OE-FOLR2 macrophages exhibit immunoregulatory and pro-angiogenic properties. **(A)** Western blot analysis (top) and quantitative analysis (bottom) of FOLR2 expression in THP-1 cells from ctrl, NC, and OE-FOLR2 groups (n = 3). Protein expression levels were quantified by densitometric analysis using ImageJ and normalized to β-actin (FOLR2/ACTB). Each Western blot experiment was performed and analyzed independently. **(B)** qRT-PCR analysis of pro-inflammatory cytokines *IL1B*, *IL6*, *TNFA*, and *CXCL10* in NC and OE-FOLR2 THP-1 cells (n = 3). **(C)** qRT-PCR analysis of immunoregulatory factors *MRC1*, *FN1*, *IL10*, and *TGFB1* in NC and OE-FOLR2 THP-1 cells (n = 3). **(D)** qRT-PCR analysis of adhesion-related molecules *ICAM1* and *ITGA4* in NC and OE-FOLR2 THP-1 cells (n = 3). **(E)** qRT-PCR analysis of pro-angiogenic factors *VEGFA* and *ANGPT1* in NC and OE-FOLR2 THP-1 cells (n = 3). **(F)** Flow cytometry analysis of CD86 expression in NC and OE-FOLR2 THP-1 cells. **(G)** Percentage of CD14^+^CD86^+^ cells in NC and OE-FOLR2 groups (n = 3). **(H)** Flow cytometry analysis of CD206 expression in NC and OE-FOLR2 THP-1 cells. **(I)** Percentage of CD14^+^CD206^+^ cells in NC and OE-FOLR2 groups (n = 3). **(J)** ELISA detection of IL-10 and TNF-α concentrations in the supernatant of NC and OE-FOLR2 THP-1 cells. **(K)** Schematic diagram of the co-culture experiment. NC and OE-FOLR2 THP-1 cells were seeded in Transwell inserts (upper chamber), while HUVECs were seeded in the lower chamber. **(L)** Tube formation assay of HUVECs on Matrigel after co-culture with NC or OE-FOLR2 THP-1 cells. Scale bar, 200 μm. **(M)** Quantification of the number of nodes, tubes, and meshes in random fields (n = 5). Data are presented as mean ± SEM. Statistical significance was determined using Student’s *t*-test or one-way ANOVA.

To investigate the functional role of FOLR2^+^ Mac, THP-1 cells were transduced with a FOLR2-overexpressing lentivirus (OE-FOLR2) or a control lentivirus (NC) and subsequently differentiated into M0 macrophages using 100 ng/mL PMA for 48 h (**Figure 4A**). qRT-PCR analysis showed that OE-FOLR2 THP-1-derived macrophages exhibited significantly reduced mRNA levels of pro-inflammatory cytokines, including *IL1B*, *IL6*, *TNFA*, and *CXCL10* (**Figure 4B**). In contrast, the expression of immunoregulatory factors *MRC1*, *FN1*, *IL10*, and *TGFB1* was markedly increased (**Figure 4C**). Furthermore, OE-FOLR2 macrophages displayed elevated expression of adhesion-related molecules *ICAM1* and *ITGA4* (**Figure 4D**), as well as pro-angiogenic factors *VEGFA* and *ANGPT1* (**Figure 4E**).

Flow cytometry analysis further demonstrated decreased expression of CD86 and increased expression of CD206, accompanied by reduced CD14^+^CD86^+^ and increased CD14^+^CD206^+^ macrophage populations in the OE-FOLR2 group (**Figures 4F–I**). Consistently, ELISA results revealed increased secretion of the anti-inflammatory cytokine IL-10 and decreased secretion of the pro-inflammatory cytokine TNF-α in OE-FOLR2 macrophages (**Figure 4J**). To further assess the pro-angiogenic function of OE-FOLR2 macrophages, HUVECs were co-cultured with NC or OE-FOLR2 macrophages for 48 h (**Figure 4K**). Tube formation assays showed that angiogenic capacity of HUVECs was significantly enhanced when co-cultured with OE-FOLR2 macrophages compared with the NC group (**Figures 4L, M**).

Collectively, these results indicate that FOLR2 overexpression suppresses inflammatory responses while promoting an immunoregulatory macrophage phenotype with enhanced adhesion and pro-angiogenic capacities, highlighting a potential role for FOLR2^+^ Mac in facilitating decidualization and vascular remodeling during early pregnancy.

### Silencing FOLR2 drives a pro-inflammatory phenotype and impairs angiogenic capacity in macrophages

**Figure 5:**
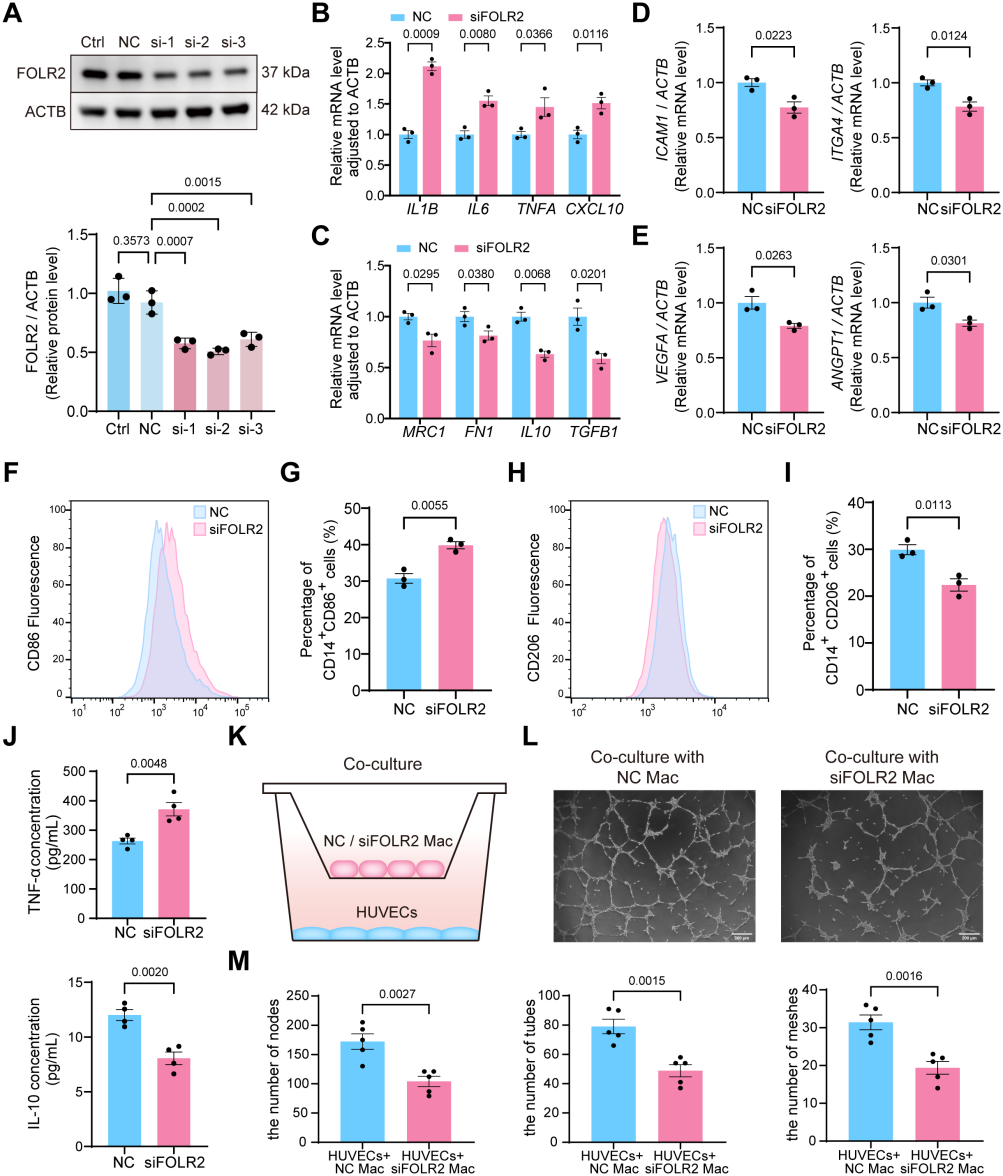
siFOLR2 macrophages exhibit increased inflammatory and reduced angiogenic properties. **(A)** Western blot analysis (top) and quantitative analysis (bottom) of FOLR2 expression in THP-1 cells from ctrl, NC, and siFOLR2 groups (n = 3). Protein expression levels were quantified by densitometric analysis using ImageJ and normalized to β-actin (FOLR2/ACTB). Each Western blot experiment was performed and analyzed independently. **(B)** qRT-PCR analysis of pro-inflammatory cytokines *IL1B*, *IL6*, *TNFA*, and *CXCL10* in NC and siFOLR2 THP-1 cells (n = 3). **(C)** qRT-PCR analysis of immunoregulatory factors *MRC1*, *FN1*, *IL10*, and *TGFB1* in NC and siFOLR2 THP-1 cells (n = 3). **(D)** qRT-PCR analysis of adhesion-related molecules *ICAM1* and *ITGA4* in NC and siFOLR2 THP-1 cells (n = 3). **(E)** qRT-PCR analysis of pro-angiogenic factors *VEGFA* and *ANGPT1* in NC and siFOLR2 THP-1 cells (n = 3). **(F)** Flow cytometry analysis of CD86 expression in NC and siFOLR2 THP-1 cells. **(G)** Percentage of CD14^+^CD86^+^ cells in NC and siFOLR2 groups (n = 3). **(H)** Flow cytometry analysis of CD206 expression in NC and siFOLR2 THP-1 cells. **(I)** Percentage of CD14^+^CD206^+^ cells in NC and siFOLR2 groups (n = 3). **(J)** ELISA detection of IL-10 and TNF-α concentrations in the supernatant of NC and siFOLR2 THP-1 cells. **(K)** Schematic diagram of the co-culture experiment. NC and siFOLR2 THP-1 cells were seeded in Transwell inserts (upper chamber), while HUVECs were seeded in the lower chamber. **(L)** Tube formation assay of HUVECs on Matrigel after co-culture with NC or siFOLR2 THP-1 cells. Scale bar, 200 μm. **(M)** Quantification of the number of nodes, tubes, and meshes in random fields (n = 5). Data are presented as mean ± SEM. Statistical significance was determined using Student’s *t*-test or one-way ANOVA.

To determine whether loss of FOLR2 alters macrophage function, THP-1 cells were transduced with NC or FOLR2-specific siRNA (siFOLR2) and subsequently differentiated into M0 macrophages using 100 ng/mL PMA for 48 h (**Figure 5A**). Knockdown of FOLR2 markedly increased the transcription of pro-inflammatory mediators, including *IL1B*, *IL6*, *TNFA*, and *CXCL10*, as determined by qRT-PCR (**Figure 5B**). In parallel, the expression of immunoregulatory genes *MRC1*, *FN1*, *IL10*, and *TGFB1* was significantly suppressed (**Figure 5C**). In addition, siFOLR2 macrophages showed reduced expression of the adhesion-related molecules *ICAM1* and *ITGA4* (**Figure 5D**), together with decreased levels of the pro-angiogenic factors *VEGFA* and *ANGPT1* (**Figure 5E**).

Flow cytometric analysis further revealed a phenotypic shift toward a pro-inflammatory state. Specifically, CD86 expression was increased, whereas CD206 expression was reduced, leading to a higher proportion of CD14^+^CD86^+^ macrophages and a lower proportion of CD14^+^CD206^+^ macrophages in the siFOLR2 group (**Figures 5F–I**). Consistent with these transcriptional changes, ELISA demonstrated elevated secretion of TNF-α and reduced secretion of IL-10 in siFOLR2 macrophages compared with the NC group (**Figure 5J**). To further evaluate the impact of FOLR2 deficiency on macrophage-mediated angiogenesis, HUVECs were co-cultured with NC or siFOLR2 macrophages for 48 h (**Figure 5K**). Tube formation assays showed that HUVEC angiogenic capacity was significantly impaired when co-cultured with siFOLR2 macrophages (**Figures 5L, M**). These findings indicate that FOLR2 silencing promotes inflammatory activation of macrophages while weakening their immunoregulatory and pro-angiogenic functions, suggesting that the reduction of FOLR2^+^ Mac in RSA may disrupt the decidual immune microenvironment, thereby impairing angiogenesis and embryo implantation.

### The crosstalk between FOLR2+ macrophages and DSCs promotes decidualization

**Figure 6:**
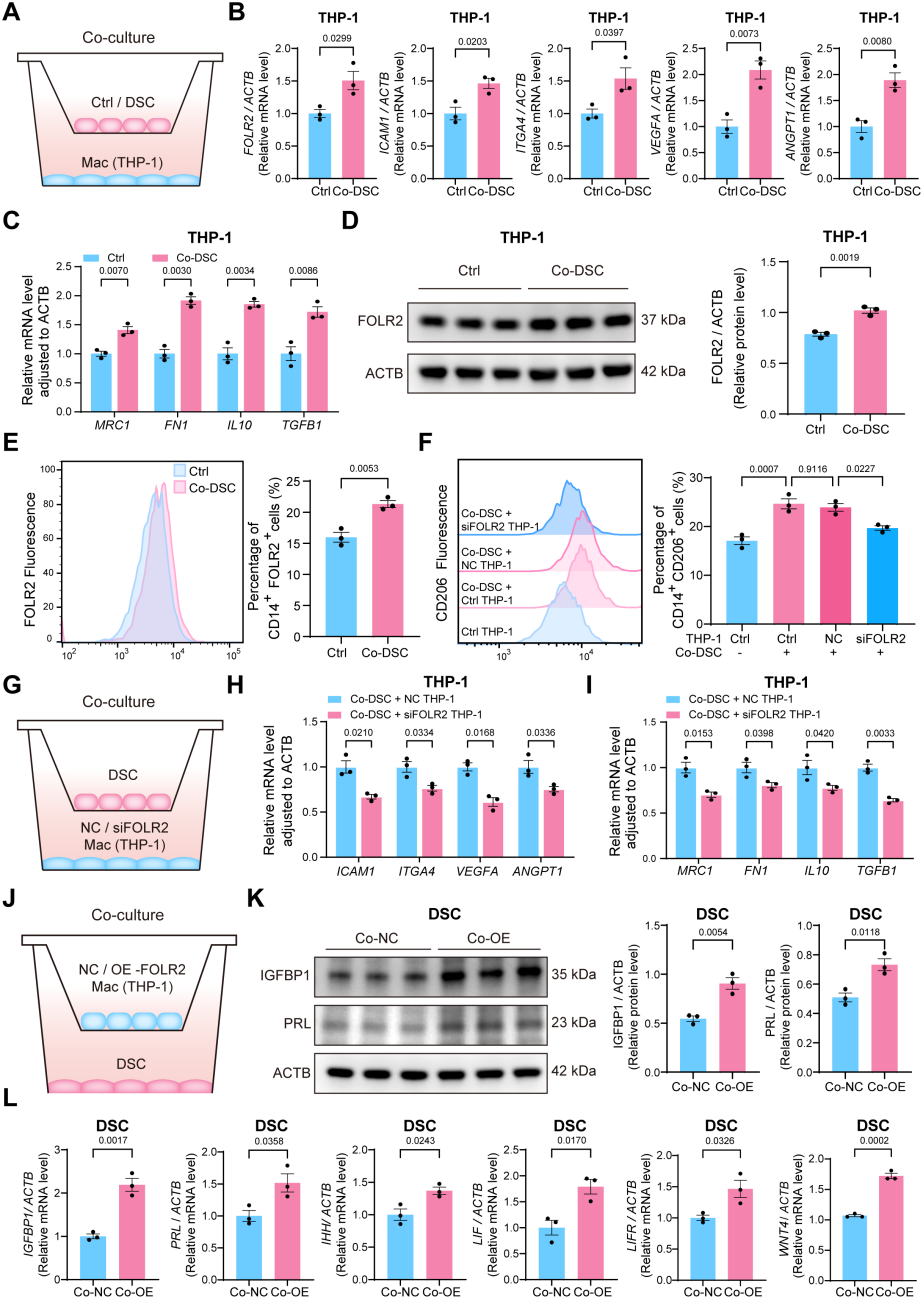
The interaction between FOLR2+ Mac and DSCs. **(A)** Schematic diagram of the DSC–Mac co-culture experiment. DSCs were seeded in Transwell inserts (upper chamber), while THP-1 cells were seeded in the lower chamber. **(B)** qRT-PCR analysis of *FOLR2*, *ICAM1*, *ITGA4*, *VEGFA* and *ANGPT1* in THP-1 cells from the control group (Ctrl) and THP-1 cells co-cultured with DSCs (Co-DSC) (n = 3). **(C)** qRT-PCR analysis of immunoregulatory factors *MRC1*, *FN1*, *IL10*, and *TGFB1* in THP-1 cells from Ctrl and Co-DSC groups (n = 3). **(D)** Western blot analysis (left) and quantitative analysis (right) of FOLR2 expression in THP-1 cells from Ctrl and Co-DSC groups (n = 3). Protein expression levels were quantified by densitometric analysis using ImageJ and normalized to β-actin (FOLR2/ACTB). **(E)** Flow cytometry analysis of FOLR2 expression (left) and the percentage of CD14^+^FOLR2^+^ cells in THP-1 cells from Ctrl and Co-DSC groups (right; n = 3). **(F)** Flow cytometry analysis of CD206 expression (left) and the percentage of CD14^+^CD206^+^ cells in THP-1 cells under Ctrl, DSC co-culture, NC siRNA + DSC co-culture, and siFOLR2 + DSC co-culture conditions (right; n = 3). **(G)** Schematic diagram of the DSC–macrophage co-culture system. DSCs were seeded in Transwell inserts (upper chamber), while NC or siFOLR2-transfected THP-1-derived macrophages were seeded in the lower chamber. **(H)** qRT-PCR analysis of *ICAM1*, *ITGA4*, *VEGFA* and *ANGPT1* in THP-1 cells co-cultured with DSCs following transfection with NC or siFOLR2 (n = 3). **(I)** qRT-PCR analysis of immunoregulatory factors *MRC1*, *FN1*, *IL10*, and *TGFB1* in THP-1 cells co-cultured with DSCs following transfection with NC or siFOLR2 (n = 3). **(J)** Schematic diagram of the Mac–DSC co-culture experiment. NC or OE-FOLR2 THP-1 cells were seeded in Transwell inserts (upper chamber), while DSCs were seeded in the lower chamber. **(K)** Western blot analysis (left) and quantitative analysis (right) of IGFBP1 and PRL expression in DSCs co-cultured with NC (Co-NC) or OE-FOLR2 (Co-OE) THP-1 cells. **(L)** qRT-PCR analysis of *IGFBP1*, *PRL*, *IHH*, *LIF*, *LIFR*, and *WNT4* in DSCs from Co-NC and Co-OE groups (n = 3). Statistical significance was determined using Student’s *t*-test or one-way ANOVA.

DSCs are pivotal for early pregnancy, as they foster immune tolerance, secrete factors essential for trophoblast invasion and angiogenesis, and remodel the extracellular matrix to maintain uterine receptivity (33). To elucidate the interaction between FOLR2^+^ Mac and DSCs, we employed an *in vitro* co-culture system (**Figure 6A**). Co-culturing THP-1-derived macrophages with DSCs robustly induced the expression of *FOLR2*, alongside key adhesion molecules (*ICAM1*, *ITGA4*) and pro-angiogenic factors (*VEGFA*, *ANGPT1*) (**Figure 6B**). This phenotypic shift was accompanied by significant upregulation of immunoregulatory genes, including *MRC1*, *FN1*, *IL10*, and *TGFB1* (**Figure 6C**). Western blotting further confirmed elevated FOLR2 protein levels (**Figure 6D**). Furthermore, flow cytometric analysis demonstrated a marked increase in the proportion of CD14^+^FOLR2^+^ and CD14^+^CD206^+^ macrophages following co-culture, underscoring their enhanced immunoregulatory capacity (**Figures 6E, F**). Importantly, co-culturing DSCs with NC or siFOLR2-transfected THP-1-derived macrophages demonstrated that knockdown of FOLR2 significantly attenuated DSC-induced upregulation of adhesion molecules, pro-angiogenic factor, and immunoregulatory genes (**Figures 6G–I**), as well as the proportion of CD14^+^CD206^+^ macrophages (**Figure 6F**), indicating that FOLR2 is required for the functional response of macrophages to DSC-derived signals.

To examine the reciprocal effects of FOLR2^+^ Mac on DSCs, we co-cultured DSCs with OE-FOLR2 THP-1-derived macrophages (**Figure 6J**). Compared with DSCs co-cultured with NC macrophages, DSCs exposed to OE-FOLR2 macrophages exhibited further upregulation of decidualization markers, including IGFBP1, PRL, IHH, LIF, LIFR, and WNT4 (**Figures 6K, L**).

Collectively, these findings suggest a potential interaction between DSCs and FOLR2^+^ Mac. DSCs promote macrophage polarization toward a FOLR2^+^ phenotype with immunoregulatory and pro-angiogenic features. Conversely, FOLR2^+^ Mac may be associated with enhanced DSC decidualization during early pregnancy.

## Discussion

**Figure 7:**
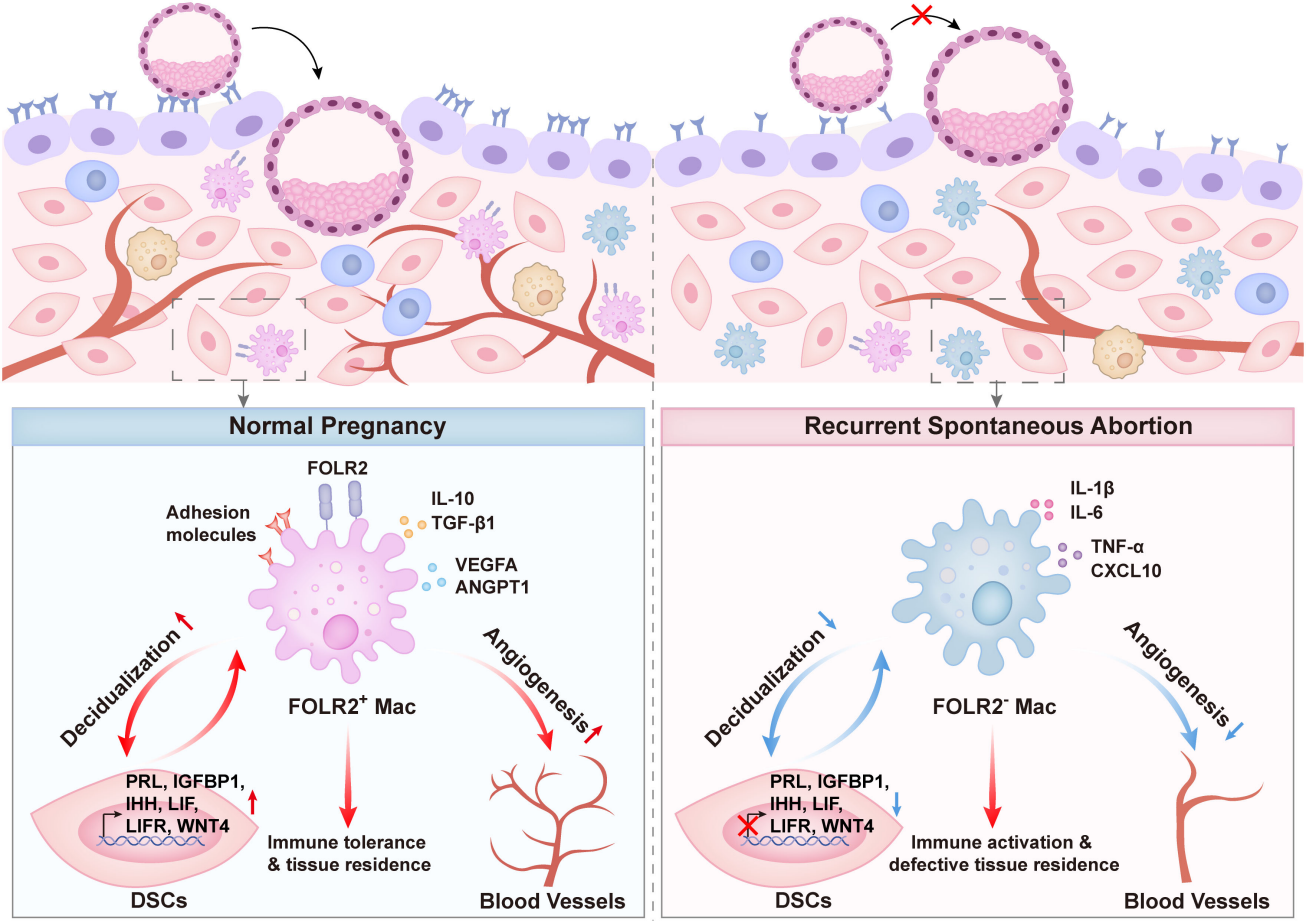
Schematic illustration of the roles of FOLR2^+^ Mac in normal pregnancy and their deficiency in RSA. During normal pregnancy, DSCs promote the recruitment of peripheral monocytes into the decidua and their differentiation into FOLR2^+^ Mac. FOLR2^+^ Mac exhibit increased expression of adhesion molecules and secrete IL-10, TGF-β1, VEGFA, and ANGPT1, thereby promoting immune tolerance, tissue residence, angiogenesis, and reinforcing the decidualization process of DSCs, which collectively facilitate endometrial receptivity and embryo implantation. In RSA, reduced abundance of FOLR2^+^ macrophages and a relative increase in FOLR2⁻ Mac, characterized by the secretion of IL-1β, IL-6, TNF-α, and CXCL10, may impair immune tolerance, angiogenesis, and decidualization, ultimately contributing to pregnancy loss.

In this study, we systematically characterized decidual macrophage heterogeneity and identified a subset of FOLR2^+^ macrophages enriched in the decidua. Through integrated single-cell transcriptomics, clinical validation, and functional experiments, we demonstrated that FOLR2^+^ macrophages exhibit immunoregulatory, tissue-resident, and pro-angiogenic properties. Notably, the reduction of FOLR2^+^ macrophages in RSA is associated with immune activation as well as impaired angiogenesis and decidualization. Mechanistically, DSCs drive the differentiation of FOLR2^+^ macrophages, which in turn reciprocally interact with DSCs to facilitate decidualization and support early pregnancy maintenance (**Figure 7**).

The decidua comprises a heterogeneous cellular landscape, including DSCs, DICs, and trophoblasts. The coordinated interactions among these cells orchestrate decidualization, immune tolerance, and angiogenesis, thereby facilitating embryo implantation and successful pregnancy. dMac predominantly exhibit an M2-like phenotype and play diverse roles in clearing apoptotic debris and promoting tissue remodeling, thereby maintaining homeostasis at the maternal–fetal interface (34, 35). Our previous study demonstrated that decidual macrophages were significantly reduced in both number and proportion in SA patients and aborted mice compared with controls (36). Moreover, compared with purified peripheral blood monocytes, dMac express more than 100 adhesion-related molecules, which facilitate their local retention within the decidua. Impaired adhesion and retention of dMac in SA further suggest that instability of this population may contribute to disease pathogenesis (36). However, the specific phenotypic characteristics and functional mechanisms of decidual macrophage subsets in SA and RSA remain largely unclear.

Through integrated single-cell transcriptomic analyses and clinical validation, we found that FOLR2^+^ Mac were markedly enriched in the decidua and predominantly exhibited an immunoregulatory phenotype characterized by CD163^+^CD206^+^ expression. FOLR2 is preferentially expressed in M2-like macrophages and has emerged as a reliable marker for identifying specific M2 macrophage subpopulations. In several cancer studies, FOLR2^+^ Mac have been characterized as a distinct subset of tissue-resident macrophages that contribute to the regulation of the local angiogenic niche and vascular remodeling (24, 25, 37). Consistently, previous single-cell studies of the human decidua also identified FOLR2 as a marker of macrophages with an anti-inflammatory phenotype (27). In line with these observations, our single-cell transcriptomic analysis revealed that FOLR2^+^ macrophages were enriched in pathways related to immune regulation, angiogenesis, cell adhesion, and TGF-β signaling. Functional experiments further demonstrated that FOLR2 overexpression in THP-1-derived macrophages enhanced immunoregulatory activity, adhesion-related molecule expression, and pro-angiogenic capacity. Together, these findings support an important role for FOLR2^+^ Mac in maintaining immune homeostasis at the maternal–fetal interface and supporting successful pregnancy, suggesting that disruption of this macrophage subset is associated with pregnancy complications such as RSA.

Traditionally, macrophage activation has been described using the M1/M2 polarization paradigm. However, accumulating evidence indicates that macrophage activation represents a continuum of transcriptional and functional states shaped by complex and dynamic microenvironmental cues, rather than a rigid binary classification (21, 22). In particular, tissue-resident macrophages at the maternal–fetal interface are highly plastic and context-dependent, exhibiting heterogeneous functional programs that extend beyond the classical M1/M2 framework (38). In this context, the M1/M2 paradigm is applied herein only as a simplified functional reference framework. Importantly, the FOLR2^+^ macrophage subset identified in this study is interpreted primarily as an immunoregulatory population based on its transcriptional signatures and functional validation, rather than being strictly equated with the conventional M2 phenotype.

At the maternal–fetal interface of RSA patients, dMac exhibit heightened pro-inflammatory activation. Specifically, dMac predominantly display a pro-inflammatory phenotype, characterized by elevated expression of CD80 and CD86 and concomitant reduction of immunoregulatory-associated markers CD163 and CD206. This phenotypic shift is accompanied by increased secretion of pro-inflammatory cytokines, including IL−1β, IL−6, and TNF−α, reflecting a shift in the M1/M2 balance toward a pro-inflammatory state in RSA (39, 40). Notably, our analysis revealed a marked reduction in both the expression of FOLR2 and the proportion of FOLR2^+^ Mac in RSA decidua, indicating a reduction in immunoregulatory macrophages. CellChat analysis suggested altered ligand–receptor interaction patterns involving FOLR2^+^ macrophages in the RSA microenvironment. Specifically, predicted interactions involving inflammatory signaling pathways, including TNF and SPP1, were enriched in FOLR2^+^ macrophages and their interacting cell populations. In contrast, ligand–receptor pairs associated with angiogenesis, immunoregulatory processes, and cell adhesion, including VEGF, TGFB, and ITGB2, showed reduced predicted interaction scores. Together, these findings suggest that FOLR2^+^ macrophage deficiency is associated with immune dysregulation at the maternal–fetal interface and impaired decidualization and embryo implantation in RSA.

Within this inflammatory context, decidual NK (dNK) cells, whose physiological functions include immune regulation and the clearance of senescent or apoptotic decidual stromal cells, are likely to undergo compensatory regulatory adaptation in response to heightened inflammatory stimulation. Specifically, we propose that the upregulation of inhibitory receptor pathways in NK cells, including the CD94/NKG2 axis (NKG2A, NKG2C, and NKG2E) as well as KLR-associated receptors (such as KLRK1, KLRC1, and KLRC2), may represent a feedback mechanism aimed at restraining excessive immune activation and maintaining local immune homeostasis. However, despite the activation of these inhibitory receptor-mediated signaling pathways, these regulatory mechanisms appear insufficient to restore immune equilibrium, as they are ultimately overwhelmed by dominant pro-inflammatory cues and broader immune network dysregulation.

DSCs play a central role in shaping the immune microenvironment at the maternal–fetal interface. Paracrine factors and extracellular matrix components derived from DSCs can induce M2-like polarization of decidual macrophages, thereby supporting immune tolerance and tissue homeostasis (41, 42). Conversely, aberrant lipid transfer from DSCs to decidual macrophages can activate pro-inflammatory pathways, linking stromal–immune metabolic crosstalk to miscarriage pathology (43). Single-cell analyses have further revealed distinct DSC–macrophage ligand–receptor interactions, with enhanced pro-inflammatory communication observed in RSA decidua (44). In our study, DSCs promoted the differentiation of macrophages into FOLR2^+^ Mac and enhanced their expression of immunoregulatory molecules, as well as factors associated with adhesion and angiogenesis, highlighting the capacity of the DSC microenvironment to drive FOLR2^+^ Mac polarization. Importantly, loss-of-function experiments further demonstrated that FOLR2 is functionally required for this process, as siFOLR2 significantly attenuated DSC-induced upregulation of immunoregulatory genes, adhesion molecules, and pro-angiogenic factors. Reciprocally, co-culture of DSCs with OE-FOLR2 macrophages increased the expression of decidualization-associated markers in DSCs, including IGFBP1, PRL, IHH, LIF, LIFR, and WNT4. Together, these findings suggest that bidirectional interactions between DSCs and FOLR2^+^ macrophages may coordinate immune homeostasis, vascular remodeling, and stromal cell decidualization, thereby contributing to the establishment of a receptive maternal–fetal interface and successful early pregnancy.

Although our study highlights the critical role of FOLR2^+^ Mac in the decidua, several limitations should be acknowledged. First, the precise molecular mechanisms and downstream signaling pathways underlying FOLR2^+^ Mac function remain incompletely understood. Importantly, although our data demonstrate a strong association between FOLR2^+^ macrophage alterations and RSA, causal relationships cannot be definitively established based on the current evidence, and further mechanistic validation will be required to strengthen causal inference. Second, THP-1-derived macrophages are widely used as a manipulable *in vitro* model, but they do not fully recapitulate the complexity of tissue-resident decidual macrophages *in vivo*. In addition, the early pregnancy decidual microenvironment is relatively hypoxic, whereas THP-1-derived macrophages were cultured under atmospheric oxygen conditions. Therefore, the *in vitro* system may not fully reflect the *in vivo* physiological microenvironment. Future studies using primary cells or *in vivo* models are required to further validate these findings under more physiological conditions. Finally, the sample size of clinical specimens was relatively limited. Although the RSA and control groups were matched for key clinical variables, heterogeneity inherent to RSA should still be considered. Validation in larger and more well-characterized cohorts will further strengthen the translational relevance of these findings.

## Conclusion

In conclusion, during normal early pregnancy, DSCs are suggested to contribute to the recruitment of peripheral monocytes into the decidua and the differentiation of FOLR2^+^ macrophages. These FOLR2^+^ macrophages are associated with immune homeostasis, driving vascular remodeling and reinforcing decidualization. The reduction of FOLR2^+^ macrophages may be associated with impaired immune tolerance, angiogenesis, and decidualization, and may be involved in the pathogenesis of RSA. FOLR2^+^ macrophages may represent potential biomarker candidates for early pregnancy viability or RSA risk assessment. Furthermore, therapeutic strategies targeting FOLR2^+^ Mac function through pharmacological or cellular approaches warrant further investigation. Additional studies in larger patient cohorts and *in vivo* models are needed to further explore the translational relevance and mechanistic basis of FOLR2^+^ Mac function in pregnancy maintenance.

## Data Availability

Publicly available datasets were analyzed in this study. This data can be found here: GEO database under accession numbers GSE183837, GSE194219, and GSE214607.
