# Supplementary material for: Immune activation induced by FOLR2+ decidual macrophage deficiency impairs decidualization and angiogenesis in spontaneous abortion
Source: Front Immunol. 2026 Jun 23;17:1852410. doi: 10.3389/fimmu.2026.1852410 (PMC13337435; doi:10.3389/fimmu.2026.1852410)
Supplement: Supplementary file 1 [file Supplementaryfile1.docx]

Supplementary Material

# Supplementary Figures and Tables

## Supplementary Figures


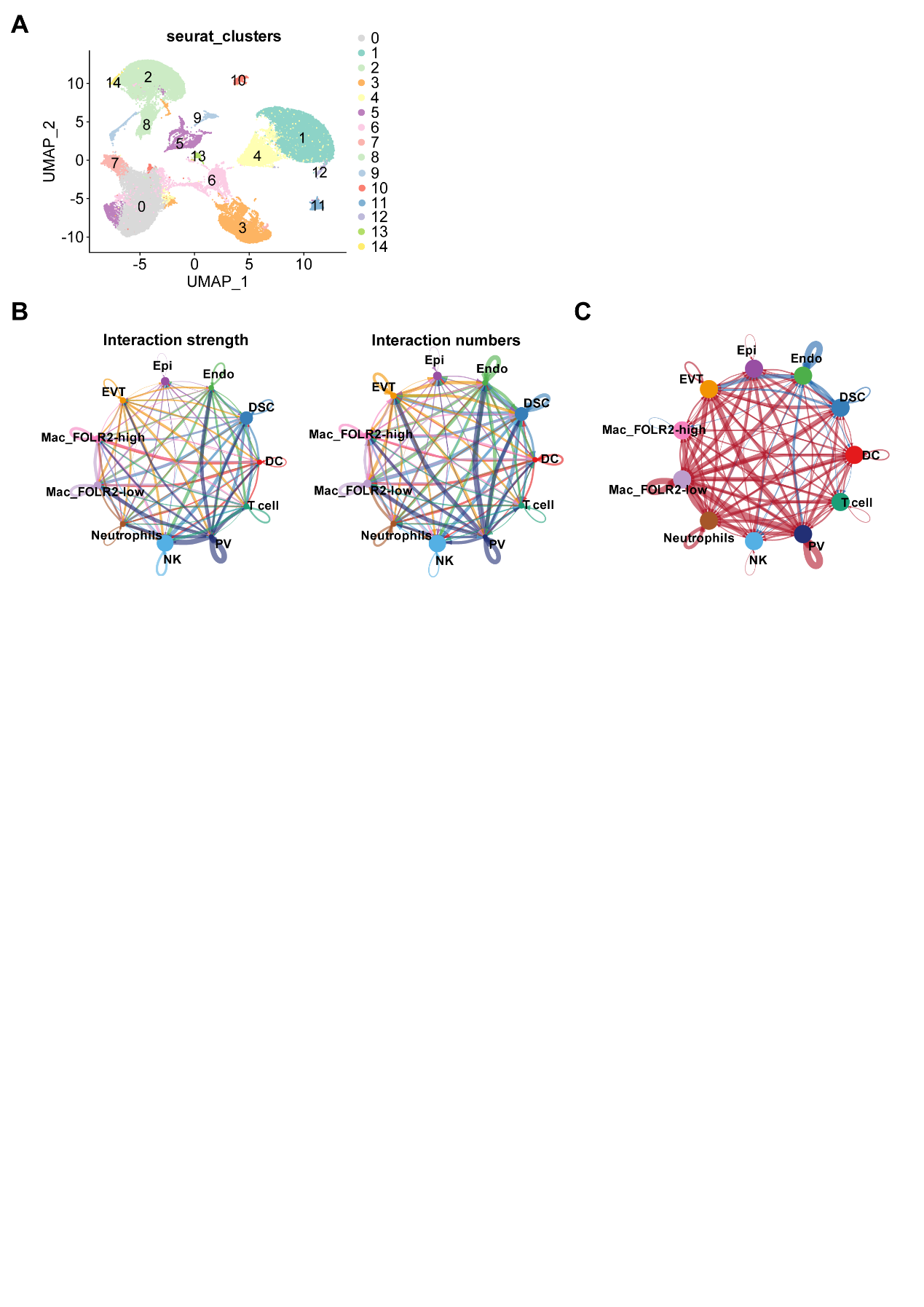


**Supplementary Figure 1.** CellChat analysis of decidual cells in normal pregnancy and RSA. (A) UMAP plot showing 15 identified cell types from single-cell transcriptomes of NP and RSA decidua. Each dot represents a single cell, colored by cell type. (B) Circle plots showing the strength and numbers of inferred interactions between different cell types in the NP and RSA groups, analyzed using CellChat. (C) Circle plots showing changes in interaction strength between different cell types. Red indicates interactions increased in the RSA group, whereas blue indicates interactions decreased in the RSA group.

**Supplementary Table 1.** Baseline characteristics of RSA patients and control subjects.

|  | Ctrl (n = 10) | RSA (n = 7) |
| --- | --- | --- |
| Age (years), mean ± SD | 30.2 ± 3.6 | 32.4 ± 2.4 |
| BMI, mean ± SD | 21.8 ± 3.8 | 20.6 ± 2.2 |
| Times of abortion, median (IQR) | 0 (0–0.3) | 2 (1.8–3.3) |
| Gestational age at termination or abortion (weeks), median (IQR) | 8 (7.4–9.2) | 8 (7.7–9.7) |
| Live births, median (range) | 1 (0-3) | 0 (0-1) |
| Menstrual cycling | Regularly (6-7 days every 28-30 days) | Regularly (6-7 days every 28-30 days) |

**Supplementary Table 2.** List of all primer sequences used for qRT-PCR.

| Gene | Forward and reverse primer |
| --- | --- |
| *IL1B* | F: 5’-ATGATGGCTTATTACAGTGGCAA-3’  R: 5’-GTCGGAGATTCGTAGCTGGA-3’ |
| *IL6* | F: 5’-ACTCACCTCTTCAGAACGAATTG-3’  R: 5’-CCATCTTTGGAAGGTTCAGGTTG-3’ |
| *TNFA* | F: 5’-CCTCTCTCTAATCAGCCCTCTG-3’  R: 5’-GAGGACCTGGGAGTAGATGAG-3’ |
| *CXCL10* | F: 5’-GTGGCATTCAAGGAGTACCTC-3’  R: 5’-TGATGGCCTTCGATTCTGGATT-3’ |
| *MRC1* | F: 5’-TCCGGGTGCTGTTCTCCTA-3’  R: 5’-CCAGTCTGTTTTTGATGGCACT-3’ |
| *FN1* | F: 5’-CGGTGGCTGTCAGTCAAAG-3’  R: 5’-AAACCTCGGCTTCCTCCATAA-3’ |
| *IL10* | F: 5’-GACTTTAAGGGTTACCTGGGTTG-3’  R: 5’-TCACATGCGCCTTGATGTCTG-3’ |
| *TGFB1* | F: 5’-GGCCAGATCCTGTCCAAGC-3’  R: 5’-GTGGGTTTCCACCATTAGCAC-3’ |
| *ICAM1* | F: 5’-ATGCCCAGACATCTGTGTCC-3’  R: 5’-GGGGTCTCTATGCCCAACAA-3’ |
| *ITGA4* | F: 5’-CACAACACGCTGTTCGGCTA-3’  R: 5’-CGATCCTGCATCTGTAAATCGC-3’ |
| *VEGFA* | F: 5’-AGGGCAGAATCATCACGAAGT-3’  R: 5’-AGGGTCTCGATTGGATGGCA-3’ |
| *ANGPT1* | F: 5’-AGCGCCGAAGTCCAGAAAAC-3’  R: 5’-TACTCTCACGACAGTTGCCAT-3’ |
| *FOLR2* | F: 5’-CCTCTGGAGTCACTCATACAAGG-3’  R: 5’-CCTGGGCTGAATCAAACCAC-3’ |
| *IGFBP1* | F: 5′-AGCACGGAGATAACTGAGGAGGAG-3′  R: 5′-GTTGGTGACATGGAGAGCCTTCG-3′ |
| *PRL* | F: 5′-GCAGATGGCTGATGAAGAGTCTCG-3′  R: 5′-GATTCGGCACTTCAGGAGCTTGAG-3′ |
| *IHH* | F: 5′-AACTCGCTGGCTATCTCGGT-3′  R: 5′-GCCCTCATAATGCAGGGACT-3′ |
| *LIF* | F: 5′-CCAACGTGACGGACTTCCC-3′  R: 5′-TACACGACTATGCGGTACAGC-3′ |
| *LIFR* | F: 5′-TGGAACGACAGGGGTTCAGT-3′  R: 5′-GAGTTGTGTTGTGGGTCACTAA-3′ |
| *WNT4* | F: 5′-GCTCTGACAACATCGCCTAC-3′  R: 5′-GCCAGCACGTCTTTAC-3′ |
| *ACTB* | F: 5’-CATGTACGTTGCTATCCAGGC-3’  R: 5’-CTCCTTAATGTCACGCACGAT-3’ |

**Supplementary Table 3.** The list of small interfering RNA (siRNA) oligos.

| Sites | Sense (5’--3’) | Antisense (3’--5’) |
| --- | --- | --- |
| Negative control | UUCUCCGAACGUGUCACGUTT | ACGUGACACGUUCGGAGAATT |
| FOLR2-si-1 | CCCGCCUGUACAACUUUAAtt | UUAAAGUUGUACAGGCGGGtt |
| FOLR2-si-2 | GGAUGUGCCCUUAUGCAAAtt | UUUGCAUAAGGGCACAUCCtt |
| FOLR2-si-3 | GGACCUCAGGAGUUAACAAtt | UUGUUAACUCCUGAGGUCCtt |
